# Neonatal Pharmacokinetics and Biodistribution of Polymeric Nanoparticles and Effect of Surfactant

**DOI:** 10.3390/pharmaceutics15041176

**Published:** 2023-04-07

**Authors:** Nuo Xu, Megan Wong, Gabrielle Balistreri, Elizabeth Nance

**Affiliations:** 1 Department of Chemical Engineering, University of Washington, Seattle, WA 98195, USA; 2 Molecular Engineering & Sciences Institute, University of Washington, Seattle, WA 98195, USA; 3 Department of Bioengineering, University of Washington, Seattle, WA 98195, USA; 4 Center for Human Development and Disability, University of Washington, Seattle, WA 98195, USA

**Keywords:** nanomedicine, pediatrics, drug delivery, half-life, nanoparticle accumulation, clinical translation

## Abstract

The development of therapeutics for pediatric use has advanced in the last few decades, yet the off-label use of adult medications in pediatrics remains a significant clinical problem. Nano-based medicines are important drug delivery systems that can improve the bioavailability of a range of therapeutics. However, the use of nano-based medicines for application in pediatric populations is challenged by the lack of pharmacokinetic (PK) data in this population. To address this data gap, we investigated the PK of polymer-based nanoparticles in term-equivalent neonatal rats. We used poly(lactic-co-glycolic acid)-poly(ethylene glycol) (PLGA-PEG) nanoparticles, which are polymer nanoparticles that have been extensively studied in adult populations but less commonly applied in neonates and pediatrics. We quantified the PK parameters and biodistribution of PLGA-PEG nanoparticles in term-equivalent healthy rats and revealed the PK and biodistribution of polymeric nanoparticles in neonatal rats. We further explored the effects of surfactant used to stabilize PLGA-PEG particles on PK and biodistribution. We showed that 4 h post intraperitoneal injection, nanoparticles had the highest accumulation in serum, at 54.0% of the injected dose for particles with Pluronic^®^ F127 (F127) as the stabilizer and at 54.6% of the injected dose for particles with Poloxamer 188 (P80) as the stabilizer. The half-life of the F127-formulated PLGA-PEG particles was 5.9 h, which was significantly longer than the 1.7 h half-life of P80-formulated PLGA-PEG particles. Among all organs, the liver had the highest nanoparticle accumulation. At 24 h after administration, the accumulation of F127-formulated PLGA-PEG particles was at 26.2% of the injected dose, and the accumulation of P80-formulated particles was at 24.1% of the injected dose. Less than 1% of the injected nanoparticles was observed in healthy rat brain for both F127- and P80-formulated particles. These PK data inform the use of polymer nanoparticle applications in the neonate and provide a foundation for the translation of polymer nanoparticles for drug delivery in pediatric populations.

## 1. Introduction

In the last few decades, the Food and Drug Administration (FDA) has encouraged the development of formulations for the pediatric population, which has resulted in more rapid advancements [1]. Specifically, both US- and European-based pediatric formulation initiatives strive to investigate nanomedicine-based formulations for pediatric use [2,3,4]. Nanomedicine has undergone explosive development in the past three decades [5,6] and is a promising therapeutic platform for pediatric populations. In addition to improving therapeutic efficacy, nanomedicine can mask drug taste, improve drug bioavailability and permeability, and reduce off-site or off-target toxicity in children [7,8,9]. Currently, most nanomedicine platforms are evaluated in adult models preclinically or in adults clinically, and off-label use of adult medications in pediatrics remains a significant clinical problem [10,11]. Off-label use of a drug can cause a higher risk of adverse drug reactions for children, especially for neonates, infants, and children younger than two years old, even though the original purpose of the off-label drug use is to benefit these patients [12,13,14].

The development of nanomedicine for pediatrics is challenged by the lack of pharmacokinetic (PK) data in the pediatric population [11,15], a gap in data that is even more significant for neonates due to the limited patient numbers and technical and ethical prerequisites of clinical trials in this patient population [10]. Even for well-established nanoparticle platforms, such as liposomes and polymersomes, PK data are limited in pediatric and neonatal populations [11]. To address this data gap, we sought to generate PK data in a neonatal model for polymer nanoparticles.

As one of the most commonly used polymeric nanoparticles, poly(lactic-co-glycolic acid)-polyethylene glycol (PLGA-PEG) nanoparticles play an important role in drug delivery due to improvements in the physicochemical and PK properties of the cargo [16,17,18]. PLGA-based formulations have been approved for several biomedical applications, such as Decapeptyl^®^, Lupron Depot^®^, Nutropin Depot^®^, Suprecur^®^ MP, Sandostatin^®^ LAR Depot, Somatuline^®^ LA, Trelstar™ Depot, Vivitrol^®^, Risperdal^®^ Consta™, etc. [19]. In addition, there are many PLGA-based formulations undergoing clinical trials, such as ciprofloxacin-loaded PLGA nanoparticles to treat *E. fecalis* infections in endodontics and quercetin-encapsulated PLGA-PEG nanoparticles (Nano-QUT) for squamous cell carcinoma treatment (NCT05456022) [20]. PLGA-based nanoformulations have good biocompatibility and have been widely studied in adults; therefore, they could have significant benefits for use in children, including neonates. However, the application of PLGA-PEG nanoparticles in neonates is still limited [21]. Here, we quantified the PK parameters and biodistribution of PLGA-PEG nanoparticles in term-equivalent healthy rats.

We also investigated the effects of using surfactant as a stabilizer in the formulation process on PK parameters and biodistribution. Prior research has revealed that surfactants used in the emulsification step have an impact on the biodistribution of polymer nanoparticles [22,23,24], yet the effects of surfactants on pediatric PK are unknown. We chose Pluronic^®^ F127 (F127) surfactant from the pluronic surfactant family and Tween^®^ 80 (P80) from the polysorbate surfactant family to formulate PLGA-PEG nanoparticles with similar particle sizes and zeta potentials. These surfactants are nonionic surfactants and are most frequently used in the field of nanomedicine due to their lower toxicity compared with ionic surfactants [25]. In addition, among nonionic surfactants, most nanoformulations include polysorbates, poly(vinyl alcohol), or Pluronics^®^ as stabilizers, emphasized by the high frequency of use in the literature [26]. Prior literature has also reported that polysorbate 80 (P80)-formulated nanoparticles have a higher affinity for apolipoprotein E (ApoE) in circulation, which is associated with enhanced brain targeting through greater blood-brain barrier (BBB) penetration [27,28,29,30,31]. Brain targeting has received increasing attention due to the difficulty of therapeutic agents in crossing the BBB. Therefore, we further focused this study on the effect of surfactant on the PK, biodistribution, and cellular association of PLGA-PEG nanoparticles in the term-equivalent brain.

Our main results provide fundamental PK data in the term-equivalent rat, which can be used to build physiologically based pharmacokinetic (PBPK) or similar models to support first-in-human predictions in neonates. Our findings also provide guidance for design and delivery of therapeutic polymeric nanoparticles for application in neonatal populations.

## 2. Materials and Methods

### 2.1. Animal Care and Ethics Statement

This study was performed in accordance with the guide for the care and use of laboratory animals of the National Institutes of Health (NIH). All animals were handled according to approved Institutional Animal Care and Use Committee (IACUC) protocols (#4383-01, approval date: 20 March 2022; #4383-02, approval date: 13 August 2020) of the University of Washington (UW), Seattle, WA. The UW has an approved Animal Welfare Assurance (#A3464-01) on file with the NIH Office of Laboratory Animal Welfare, is registered with the United States Department of Agriculture (certificate #91-R-0001), and is accredited by AAALAC International. Time-mated pregnant female Sprague–Dawley rats (virus antibody-free CD^®^ (SD) IGS, Charles River Laboratories, Raleigh, NC, USA) were purchased and arrived on postnatal day 5 (P5) with a litter of 10 sex-balanced pups. Dams were housed individually with their litter and allowed to acclimate to their environment. Before and after the experiment, each dam and her pups were housed under standard conditions with an automatic 12 h light/dark cycle, a temperature range of 20–26 °C, and access to standard chow and autoclaved tap water ad libitum. The pups were checked for health daily.

### 2.2. Polymer Activation and Labeling

PLGA-PEG (45k:5k, LA:GA = 50:50, Akina, Inc., West Lafayette, IN, USA) polymer was covalently labeled with fluorescent dye before nanoparticle formulation. To activate the PLGA-PEG polymer, the polymer was dissolved in a 20 mL glass scintillation vial with dichloromethane (DCM, Fisher Scientific, Pittsburgh, PA, USA) to create a 100 mg/mL polymer solution. P-nitrophenyl chloroformate (PNCF, Sigma-Aldrich, St. Louis, MO, USA) was dissolved in DCM to create a 10 mg/mL stock solution. PNCF solution was added to the polymer solution, followed by the immediate addition of pyridine (Sigma-Aldrich). The solution was stirred and reacted for 3 h at 200–300 rpm, and then the polymer reaction solution was slowly added to cold ethyl ether (Sigma-Aldrich) to stop the reaction. The solution was then centrifuged at 1000× *g* for 2 min and lyophilized overnight to dry the activated polymer. After polymer drying, CF647 Succinimidyl Ester (CF647^®^, Biotium, Fremont, CA, USA) was dissolved in dimethylformamide (DMF, Fisher Scientific) to make a 2 mg/mL stock solution. Activated polymer was dissolved in DMF, and then the CF647^®^ solution was added to the polymer solution. Triethylamine (TEA, Sigma-Aldrich) was added immediately after CF647 and reacted for 4 h. The solution was lyophilized overnight to dry the dye-labeled polymer. CF647-labeled polymer was stored at −20 °C for future use.

### 2.3. Nanoparticle Preparation and Characterization

Nanoparticles were prepared using the standard nanoprecipitation method, as described previously [21]. Briefly, 20 mg PLGA-PEG was dissolved in 1 mL acetone (Sigma-Aldrich) to prepare a 20 mg/mL organic solution, and then this solution was added dropwise into 25 mL of 1% (*v*/*w*) P80 or 1% (*w*/*w*) F127 solution. The nanoparticles (PLGA-PEG/F127 and PLGA-PEG/P80) formed spontaneously in the surfactant solution. The solution was stirred magnetically at 500 rpm for 3 h, and any remaining organic solvent was removed by rotary evaporation at 4 °C under reduced pressure for 30 min. After that, nanoparticles were collected at 100,000× *g* for 60 min and washed at 100,000× *g* for 25 min by centrifugation. Nanoparticles were resuspended in 1× PBS after washing and then stored at 4 °C for future use.

The particle size and polydispersity index (PDI) of nanoparticles were measured using dynamic light scattering (DLS), and the zeta potential (ζ-potential) was determined using a zeta potential analyzer (NanoSizer Zeta Series, Malvern Instruments, Malvern, UK). Samples were diluted to appropriate concentrations to obtain accurate measurements in 10 mM sodium chloride (NaCl, Sigma-Aldrich), pH 7.0. Samples were also analyzed via transmission electron microscopy (TEM).

### 2.4. Transmission Electron Microscopy Method

To prepare nanoparticles for TEM, the samples were diluted to a 1:500 ratio (sample to DI water) from a batch concentration of 20 mg/mL. PLGA nanoparticles served as a control. PLGA-PEG and PLGA in DI water, 1% F127, and 1% P80 were adsorbed on a 200-mesh carbon film grid (Fisher Scientific, Hampton, NH, USA), negatively stained with uranyl acetate (Ted Pella Inc., Redding, CA, USA), and then washed in DI water. Samples were prepared and imaged on the Tecnai F20 SuperTwin TEM instrument at the Molecular Analysis Facility (MAF) at the University of Washington.

### 2.5. Nanoparticle Administration and Tissue Extraction

Healthy rats (n = 4 per timepoint; 2F and 2M per timepoint) at P10 (term-equivalent to humans) received intraperitoneal (i.p.) injections of CF647-labeled PLGA-PEG/F127 or PLGA-PEG/P80 nanoparticles at a PLGA-PEG concentration of 150 mg/kg rat in 1× PBS. Tail veins are not accessible in this age of rat; therefore, i.p. was the chosen route of administration. Animals were returned to the dams after receiving a single dose of nanoparticles. Rats were sacrificed at the indicated time (30 min, 1 h, 4 h, 8 h, 24 h, 72 h) after injection by an overdose of euthanasia solution. For each rat, the brain, heart, lungs, liver, spleen, kidneys, and blood were collected. Blood was collected in a heparin-coated 1.5 mL tube, and serum was separated via 5 min of centrifugation at 3000× *g*.

### 2.6. High-Performance Liquid Chromatography (HPLC) Method

To verify the stability of the CF647-labeling on PLGA-PEG/F127 and PLGA-PEG/P80 after i.p. injection, CF647-labeled PLGA-PEG/F127 and PLGA-PEG/P80 nanoparticles were i.p. injected into P10 rats. The rats were sacrificed 4 h after the administration to collect blood. Serum extracts were collected using an Amicon filter tube (Ultracel^®^ membrane, 3 kDa cutoff) to filter serum proteins. Free CF647, serum extract from whole blood (blank sample), and serum extracts from blood from nanoparticle-injected rats were analyzed using a C18-based column (Agilent Eclipse XDB-C18, 150 mm × 4.6 mm, 5 μm). The mobile phase consisted of 90% acetonitrile and 10% 10 mM aqueous ammonium acetate (pH 7.5 before mixing). The column temperature was set to 30 °C. The flow rate was 0.5 mL/min, and the injection volume was 10 μL. Absorbance detection was performed by UV at 650 nm, and the total run time was 10 min.

### 2.7. Tissue Processing for PK and Organ-Level Biodistribution Analysis

To quantify the PK profiles and organ-level biodistribution, one hemisphere of the brain, one lobe each of the lung and liver, one kidney, and the heart and spleen were homogenized in 1× PBS, and the supernatants were collected via centrifugation at 10,000× *g* for 10 min. The PK parameters of PLGA-PEG/F127 and PLGA-PEG/P80 in serum, brain, heart, lung, liver, spleen, and kidney tissues were calculated from a calibration curve for each tissue using UV-Vis spectrometry. Tissue from pups not injected with nanoparticles served as controls. The noncompartmental PK analysis method was used to determine the PK parameters in P10 rat serum, brain, and other organs, as described elsewhere [32]. A graphical relationship between PLGA-PEG concentration (y-axis) and time (x-axis) was established. Then, the data points from the terminal elimination phase were used to calculate the elimination rate constant (*K_e_*).

(1) $$K_{e}=\frac{lnC_{1}-lnC_{2}}{t_{2}-t_{1}}$$

*C*_1_ and *C*_2_ are the concentrations of PLGA-PEG in the terminal elimination phase, and *t*_1_ and *t*_2_ are the corresponding times, respectively.

The area under the PLGA-PEG concentration versus time curve (AUC) from time zero (*C*_0_) until 72 h (AUC_(0-72)_) was calculated using GraphPad Prism Version 9.4.1 (GraphPad Software Inc., San Diego, CA, USA). Because no to minimal PLGA-PEG polymer was detectable at 72 h, the total AUC was defined as:

(2) $$\mathrm{AUC}={\mathrm{AUC}}_{\left(0\text{-}72\right)}$$

Volume of distribution (*V_d_*) and volume of distribution at steady state were calculated as:

(3) $$V_{d}=\frac{\mathrm{Injected}\;\mathrm{dose}}{\mathrm{AUC}\times K_{e}},$$

and

(4) $$V_{ss}=\mathrm{Cl}\times \mathrm{MRT}$$

Clearance (Cl) was calculated as:

(5) $$\mathrm{Clearance}=\frac{\mathrm{Injected}\;\mathrm{dose}}{\mathrm{AUC}}$$

Elimination half-life (*T*_1/2_) was calculated as:

(6) $$T_{1/2}=\frac{0.693}{K_{e}}$$

The area under the moment of PLGA-PEG concentration (PLGA-PEG × time) versus time curve (AUMC) from time zero until 72 h (AUMC_(0-72)_) was calculated using GraphPad Prism. Similarly, total AUMC was defined as:

(7) $$\mathrm{AUMC}={\mathrm{AUMC}}_{\left(0\text{-}72\right)}$$

The mean residence time (MRT) of PLGA-PEG was calculated as:

(8) $$\mathrm{MRT}=\frac{\mathrm{AUMC}}{\mathrm{AUC}}$$

### 2.8. Immunohistochemistry

To characterize the tissue-level biodistribution, one hemisphere of the brain, one portion each of the lung and liver, and one kidney were placed in a formalin-to-30% sucrose gradient and cryosectioned on a Leica cryostat into 30 μm sections [21]. To evaluate brain distribution, primary antibodies for microglia (1:250 rabbit anti-Iba1, Wako, Fujifilm, Minato City, Tokyo, Japan) and neurons (1:250 donkey anti-NeuN, Abcam, Cambridge, UK) were prepared in 1× PBS containing 1% Triton X-100 (Sigma-Aldrich) and 3% normal goat serum (Sigma-Aldrich). Primary antibody solutions were added to tissue sections for 4–6 h at room temperature in a humified dark chamber. The tissue slices were washed twice in 1× PBS. Secondary antibodies were dissolved in 1× PBS containing 1% Triton X-100 and added to the tissue slices with 2 h incubation. The slices were washed twice in 1× PBS, then stained with 1:10,000 4′,6-diamidino-2-phenylindole (DAPI, Invitrogen, Waltham, MA, USA). The slides were washed and dried for 30 min in the dark. Mounting medium (Dako, Agilent Technologies, Santa Clara, CA, USA) was added to each slide, and a glass coverslip was placed on top. Slides were stored at 4 °C until imaged under an A1 confocal microscope (Nikon, Tokyo, Japan) and at −20 °C for long-term storage. For other organs, tissue sections were stained with DAPI and imaged using confocal microscopy.

### 2.9. Statistical Analysis

Statistical analysis was performed using Welch’s *t*-test. All statistical analyses were carried out using GraphPad Prism (GraphPad Software Inc., Version 9.4.1). A *p*-value of <0.05 was considered statistically significant. Calculated PK parameters are reported as mean ± standard deviation in all tables.

## 3. Results

### 3.1. Characterization and Stability of CF647-Labeled Nanoparticles

**Figure 1:**
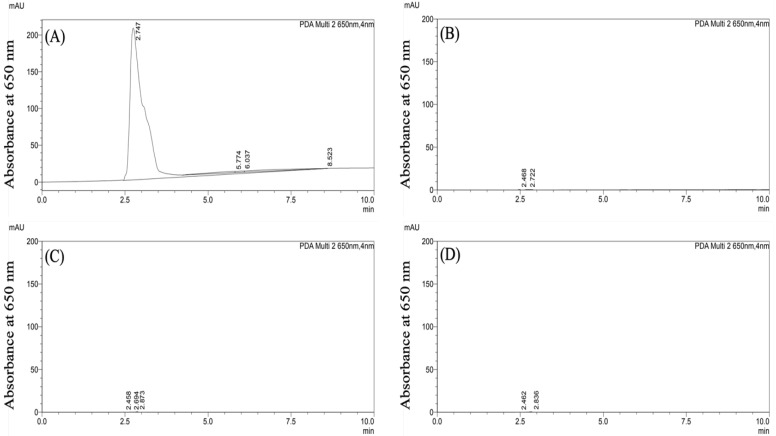
Representative HPLC chromatograms acquired based on absorbance at 650 nm for: (**A**) free CF647 dye at 20 nmol/mL concentration, (**B**) blank serum extracts, (**C**) serum extracts obtained from rats 4 h post injection of PLGA-PEG/F127 at a 150 mg/kg dosage, and (**D**) serum extracts obtained from rats 4 h post injection of PLGA-PEG/P80 at a 150 mg/kg dosage.

CF647-labeled PLGA-PEG nanoparticles were formulated in 1% F127 surfactant and 1% P80 surfactant via the standard nanoprecipitation method. Following formulation, hydrodynamic size, PDI, and zeta potential were determined using DLS and Zetasizer (Table 1). The two PLGA-PEG formulations had similar physicochemical properties: PLGA-PEG/F127 nanoparticles were 60.2 ± 0.8 nm, −2.6 ± 0.3 mV, and had a PDI of 0.2 ± 0.01. PLGA-PEG/P80 nanoparticles were 66.2 ± 1.4 nm, −2.0 ± 0.3 mV, and had a PDI of 0.2 ± 0.01. The stability of the CF647 dye-labeled nanoparticles was verified indirectly due to limited ability to visualize polymeric nanoparticles directly in tissue samples [33]. Detected at 650 nm absorbance, free CF647 dye had an absorbance peak at 650 nm (Figure 1A). The blank serum sample showed no absorbance signal at 650 nm (Figure 1B). Serum extracts obtained from rats 4 h post i.p. injection of PLGA-PEG/F127 and PLGA-PEG/P80 nanoparticles (Figure 1C,D) showed no absorbance peaks, which indicates that there was no detectable free CF647 dye in the serum following i.p. injection. No peak was identified with a fluorescence detector at Ex/Em 635 nm/665 nm (Figure S1) for the serum samples containing particles as well. These results suggest that CF647-labeled PLGA-PEG nanoparticles retain the CF647 label after i.p. injection. The colloidal stability of these particles in serum has previously been shown [24].

### 3.2. Characterization of Nanoparticles on TEM

**Figure 2:**
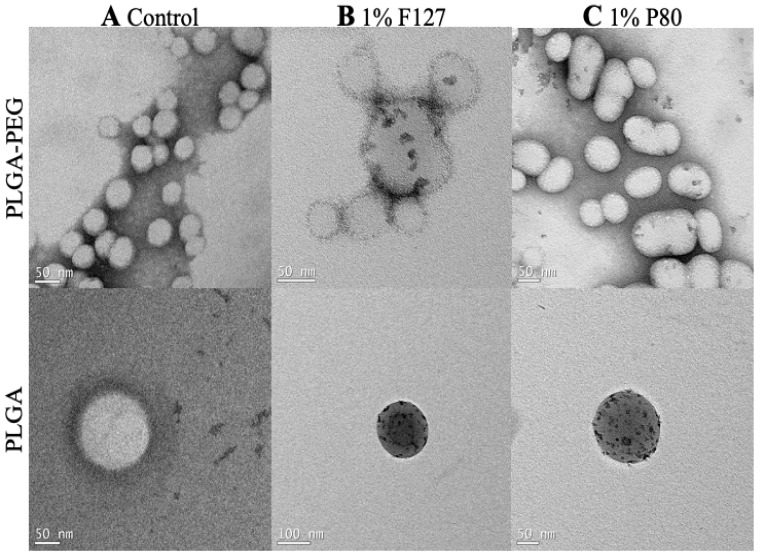
TEM images of PLGA-PEG and PLGA polymers with 1% F127 and 1% P80 surfactant solutions. (**A**) Controls are nanoparticles fabricated without surfactant solutions, (**B**) nanoparticles in 1% F127 solution, and (**C**) nanoparticles in 1% P80 solution. Scale bars: 50 nm and 100 nm.

The TEM images display the PLGA and PLGA-PEG nanoparticles with and without surfactants. Nanoparticles and surfactant can be distinguished by color contrast, which can be important for identifying surfactant to nanoparticle surface association. The control nanoparticles (Figure 2A) are PLGA and PLGA-PEG nanoparticles without surfactant used during the nanoprecipitation process. These images show the nanoparticles without electron-dense regions on the surface. The 1% F127 and 1% P80 nanoparticles (Figure 2B,C) are PLGA and PLGA-PEG nanoparticles with 1% F127 and 1% P80 surfactants used during the nanoprecipitation process. These images show nonuniform electron-dense regions on the surface of the nanoparticles, where the electron-dense regions represent F127 and P80 on the surface of the nanoparticles after formulation, collection, and washing.

### 3.3. PK and Biodistribution of PLGA-PEG in Term-Equivalent Rats

**Figure 3:**
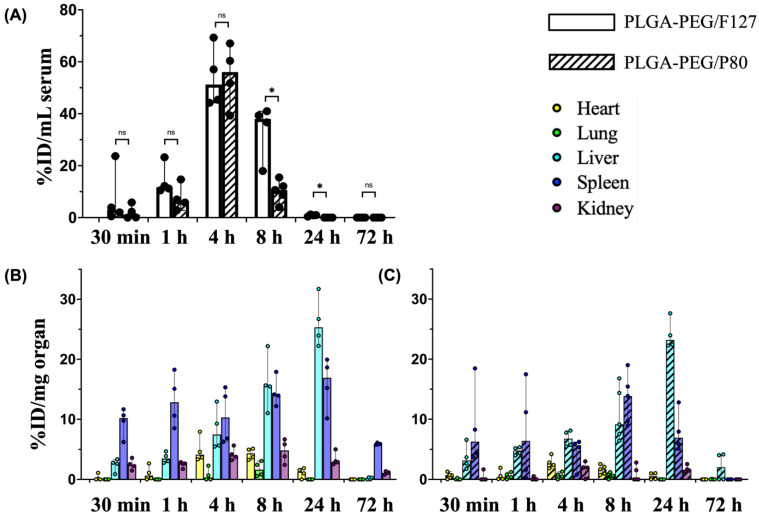
Biodistribution of PLGA-PEG/F127 and PLGA-PEG/P80 versus post-injection time (30 min, 1 h, 4 h, 8 h, 24 h, and 72 h) in P10 rats. (**A**) Serum and (**B**,**C**) organs (heart, lung, liver, spleen, and kidney) after i.p. injection (n = 4 per timepoint), %ID: percentage of injected dose. Graphs display median ± 95% confidence interval. Group differences were evaluated using Welch’s *t*-test; ns: not significant; *: *p* < 0.05.

Both PLGA-PEG/F127 and PLGA-PEG/P80 nanoparticles entered systemic circulation within 30 min after i.p. injection, reached the maximum serum concentration at approximately 4 h, and were not detectable at 72 h (Figure 3A). At the maximum serum concentration, 54.0% of the injected PLGA-PEG/F127 nanoparticles and 54.6% of the injected PLGA-PEG/P80 nanoparticles were detected in serum, showing no significant difference between these formulations. However, compared with PLGA-PEG/P80 nanoparticles, PLGA-PEG/F127 nanoparticles had a longer half-life, larger volume of distribution, longer mean residence time, and slower clearance rate in the systemic circulation (Table 2). The maximum serum concentration of PLGA-PEG/F127 nanoparticles was 2.1 mg/mL, which was lower than PLGA-PEG/P80 nanoparticles (2.3 mg/mL), but this difference was not significant. At 8 h post administration, the PLGA-PEG/F127 concentration was significantly higher than that of PLGA-PEG/P80. At 24 h after injection, no PLGA-PEG/P80 nanoparticles were detectable via UV-Vis spectrometry, but PLGA-PEG/F127 nanoparticles remained detectable in some rats, albeit at relatively low concentrations. No F127- or P80-formulated nanoparticles were detectable 72 h after i.p. injection.

For both PLGA-PEG/F127 and PLGA-PEG/P80 nanoparticles, the heart, lung, and liver reached the maximum nanoparticle concentrations at approximately 4 h, 8 h, and 24 h (Table 3) after i.p. injection, respectively. PLGA-PEG/F127 accumulation in the spleen reached the maximum concentration by 24 h after administration, but PLGA-PEG/P80 had the maximum accumulation by 8 h post injection. PLGA-PEG/F127 accumulation reached the peak in the kidney by 8 h after i.p. injection, but the maximum concentration of PLGA-PEG/P80 occurred by 24 h. In the heart, lung, spleen, and kidney, PLGA-PEG/F127 had a longer half-life than PLGA-PEG/P80—the only exception where PLGA-PEG/F127 had a shorter half-life than PLGA-PEG/P80 was in the liver. PLGA-PEG/F127 had a shorter mean residence time in the lung and liver, while PLGA-PEG/P80 had a shorter mean residence time in the heart, spleen, and kidney.

Collectively, PLGA-PEG/F127 had a higher maximum concentration and slower clearance rate compared with PLGA-PEG/P80. Following i.p. injection, PLGA-PEG/F127 appeared in the liver, spleen, and kidney in the first 30 min and appeared in the heart in the first 1 h. At 72 h, no to minimal concentrations were detected in the heart, lung, and liver (Figure 3B). In the lung, PLGA-PEG/F127 was detected only between 4 h and 8 h. PLGA-PEG/P80 was first detectable in the heart, liver, and spleen approximately 30 min after i.p. injection and became detectable in the lungs and kidney 1 h after injection. At 24 h, except in the lung, PLGA-PEG/P80 was still detectable in the heart, liver, spleen, and kidney, but there were no to minimal concentrations detected at 72 h (Figure 3C). Among all organs for both formulations, the liver accumulated the most nanoparticles (26.2% injected PLGA-PEG/F127 at 24 h, 24.1% injected PLGA-PEG/P80 at 24 h), whereas the heart, lung, and kidney had relatively low accumulations (less than 5% injected dose), and the spleen accumulated intermediate amounts at 16.0% injected dose PLGA-PEG/F127 at 24 h and 13.4% injected dose PLGA-PEG/P80 at 8 h.

### 3.4. Tissue-Level Biodistribution

**Figure 4:**
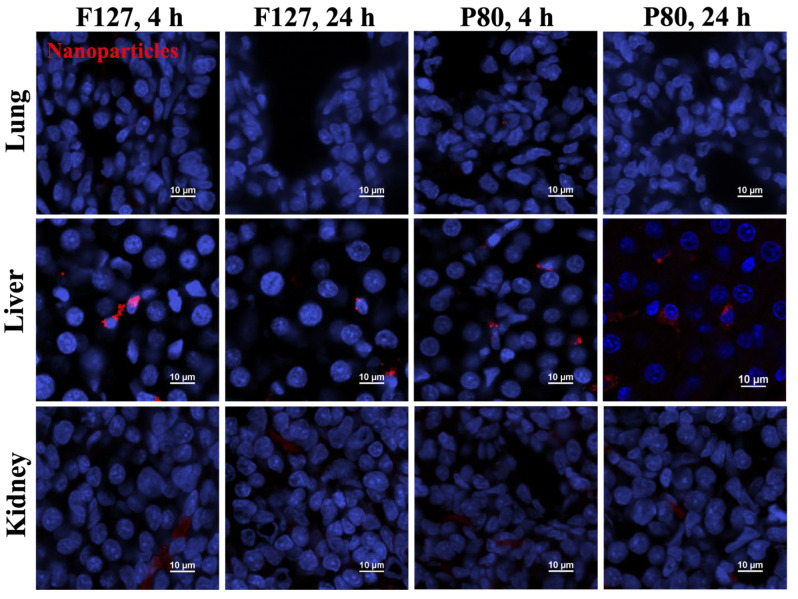
Tissue-level biodistribution of PLGA-PEG/F127 (F127) and PLGA-PEG/P80 (P80) nanoparticles (red) in P10 rat lung (**first row**); liver (**second row**); kidney (**third row**) at 4 h (**first and third column**) and 24 h (**second and fourth column**) after administration. Blue: DAPI nuclei stain. Scale bars: 10 μm.

To further explore the nanoparticle distribution in tissue within a single organ, tissues from the lung, liver, and kidney were cryosectioned and stained, and then tissue-level biodistribution was characterized using confocal microscopy at 240× magnification (Figure 4). No obvious differences in biodistribution were noticed between PLGA-PEG/F127 and PLGA-PEG/P80 in the lung, liver, and kidney at both 4 h and 24 h after administration. Following i.p. injection, few nanoparticles were observed in P10 rat lung at 4 h, and no nanoparticles were observed at 24 h. As the organ with the highest nanoparticle accumulation, many nanoparticles were observed in the liver at both 4 h and 24 h after administration. In the kidney tissue, all the nanoparticles were found in blood vessels, and no nanoparticles were observed in the tissue parenchyma. Nanoparticles were not localized within nuclei, as evidenced by a lack of colocalization between the DAPI nuclear stain and the particles in any tissue.

### 3.5. PLGA-PEG PK and Biodistribution in the Term-Equivalent Brain

**Figure 5:**
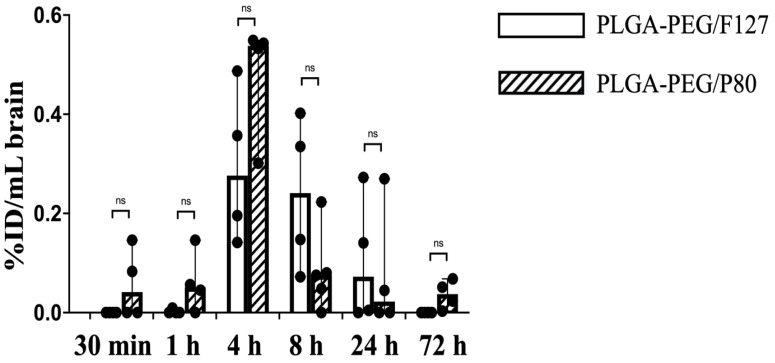
Biodistribution of PLGA-PEG/F127 and PLGA-PEG/P80 versus post injection time (30 min, 1 h, 4 h, 8 h, 24 h, and 72 h) in P10 rat brain after i.p. injection (n = 4 per timepoint). Graph displays median ± 95% confidence interval. Group differences were evaluated using Welch’s *t*-test; ns: not significant.

There are many neonatal nanotherapeutic applications in which the brain is the target organ for nanoparticle delivery, such as hypoxic-ischemic encephalopathy (HIE, which occurs in 1–8 per 1000 livebirths in developed countries), acute seizures (the most common neurological emergency in newborn babies, arising in approximately 3 per 1000 term livebirths), and periventricular leukomalacia (PVL, which affects 3–4% of preterm newborns) [34,35,36]. Neonatal brain injury, regardless of etiology, has no cure, and many brain injuries around birth or early in life have long-term effects, even if clinical treatment is provided. In addition, drug delivery to the brain is an ongoing challenge. Given the promise of prior work with PLGA-PEG nanoparticles for delivery to the injured newborn brain [21,37], we further explored brain PK and biodistribution. Herein, PLGA-PEG/F127 nanoparticles in P10 rat brain were not detectable before 1 h and after 72 h, and reached the maximum concentration by 4 h post injection. PLGA-PEG/P80 nanoparticle concentration in the P10 rat brain started increasing at 30 min after injection, reached the maximum brain concentration around 4 h, and was still detectable in some pups 72 h after i.p. injection (Figure 5). At the maximum brain concentration, PLGA-PEG/P80 concentration (0.02 mg/mL) was higher than PLGA-PEG/F127 concentration (0.01 mg/mL), but both formulations had low brain accumulation (less than 1% of injected dose), which is consistent with other literature, especially given that these were healthy animals [38,39]. PLGA-PEG/P80 nanoparticles had a shorter half-life, higher clearance rate, and lower volume of distribution compared with PLGA-PEG/F127 nanoparticles. It is worth noting that PLGA-PEG/P80 nanoparticles had a longer mean brain residence time but a shorter brain half-life (Table 4).

**Figure 6:**
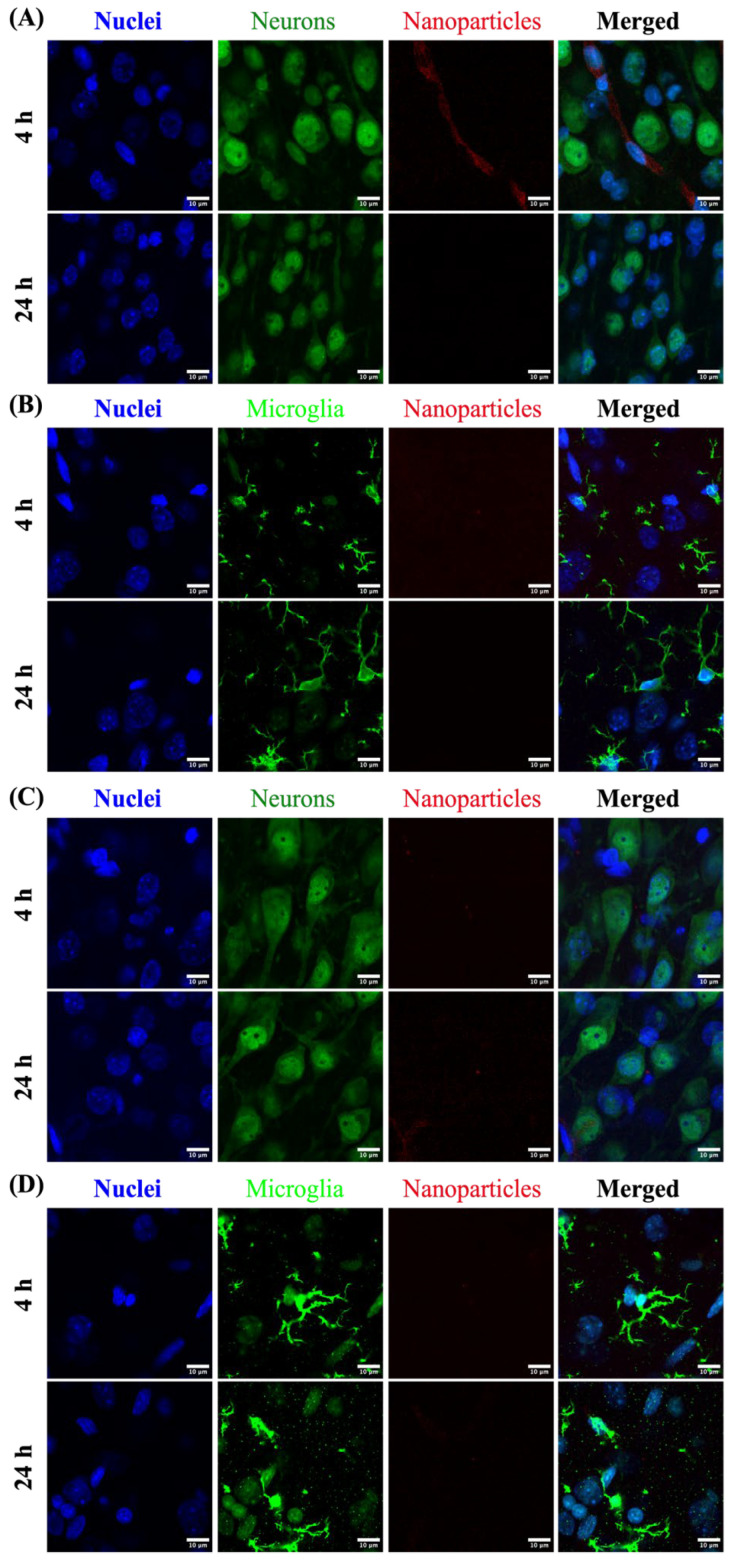
Localizations of PLGA-PEG/F127 and PLGA-PEG/P80 nanoparticles (red) in the cortex of healthy P10 rat brain at 4 h and 24 h after administration. (**A**) PLGA-PEG/F127 in cortical neurons, (**B**) PLGA-PEG/F127 in cortical microglia, (**C**) PLGA-PEG/P80 in cortical neurons, and (**D**) PLGA-PEG/P80 in cortical microglia. Blue: DAPI nuclei stain. Scale bars: 10 μm.

In the cortex of the brain, the localization of PLGA-PEG/F127 and PLGA-PEG/P80 were also different. Small amounts of PLGA-PEG/P80 nanoparticles were observed at both 4 h and 24 h in the extracellular space adjacent to cortical neurons (Figure 6C). However, for PLGA-PEG/F127 nanoparticles, at both 4 h and 24 h after i.p. injection, no to minimal PLGA-PEG/F127 nanoparticles were found in the extracellular space (Figure 6A,B). No PLGA-PEG/F127 or PLGA-PEG/P80 nanoparticles were observed to be internalized within neurons or microglia in the cortex.

## 4. Discussion

PK and biodistribution of nanoparticles depend on surface functionality, composition, particle size, surface charge, and particle shape [40,41,42]. It is necessary to control the physicochemical properties of nanoparticles when comparing the effect of surfactant. In this study, both PLGA-PEG/F127 and PLGA-PEG/P80 were formulated to produce particles within a 60–70 nm size range, 0.2 PDI, and near neutral zeta potential, which minimized the impact of physicochemical property differences. At 4 h post injection, 54.0% of the injected dose of PLGA-PEG/F127 and 54.6% of the injected dose of PLGA-PEG/P80 were detected in rat serum. PLGA-PEG/F127 and PLGA-PEG/P80 had a similar time delay to enter the systemic circulation, underwent a rapid concentration increase in serum since 1 h, and reached peak concentration at 4 h. No significant difference was found between these two formulations during the absorption process. Although the biodistribution of a nanoformulation can be impacted by its surface functionality, composition, surface charge, and particle shape, the size of nanoparticles is critical to the biodistribution of nanoparticles. The percentage of injected dose in serum can vary when there is a 20 nm size difference for a nanoparticle [43,44,45]. Generally, smaller nanoparticles need a shorter time for absorption from the peritoneal cavity into the systemic circulation, which results in higher bioavailability but shorter exposure time compared with larger nanoparticles [46]. The similar sizes of PLGA-PEG/F127 and PLGA-PEG/P80 may explain the similarity in the absorption processes of these two formulations. The systemic half-life of PLGA-PEG/F127 (5.9 h) was significantly longer than PLGA-PEG/P80 (1.7 h), which indicates that PLGA-PEG/F127 had a slower elimination in serum. P80 is reported to have higher affinity for ApoE [27,29,47]. Therefore, one potential explanation for the different profiles is that the protein composition of the corona adsorbed on PLGA-PEG/P80 is different from that of PLGA-PEG/F127. Overall, the bioavailability of PLGA-PEG/F127 was higher than PLGA-PEG/P80, which is consistent with their area under the curve (23.1 h*mg/mL for PLGA-PEG/F127 and 12.4 h*mg/mL for PLGA-PEG/P80). From the perspective of serum half-life and bioavailability, compared with PLGA-PEG/P80, PLGA-PEG/F127 can enhance systemic circulation time, which can prolong the dosing interval.

At the organ level, PLGA-PEG/F127 had a longer half-life in the heart, lung, spleen, and kidney, which followed the same trend as that of serum. In contrast, PLGA-PEG/P80 had a longer liver half-life. This may also be caused by the higher ApoE composition adsorbed on PLGA-PEG/P80 nanoparticles, as both low density lipoprotein (LDL) and LDL receptor-related protein (LRP) receptors are highly expressed in liver tissue [28,31]. For all organs, PLGA-PEG/F127 had a higher area under the curve than PLGA-PEG/P80, although the difference was not significant. This biodistribution data can be explained by the shorter systemic half-life and lower bioavailability of PLGA-PEG/P80. For both PLGA-PEG/F127 and PLGA-PEG/P80, nanoparticle accumulation was highest in the liver and spleen of the neonatal rats, which is consistent with prior literature for polymer nanoparticle delivery in adult rodents [41,45]. Due to the immaturity of their metabolic system, at the same dosage, term neonates are more sensitive than adults to some active agents [48]. Hence, when using PLGA-PEG nanoparticles in the 60–70 nm size range for drug delivery in neonatal rats, cytotoxic evaluation should focus on the liver and spleen.

For brain-specific targeting, it is notable that PLGA-PEG/P80 nanoparticles were still detectable 72 h after administration, but PLGA-PEG/F127 nanoparticles were not observed in the brain parenchyma at 24 h (Figure 6). This trend is also supported by the longer mean residence time of PLGA-PEG/P80. The contrast in the half-life, area under the curve, and mean residence time might be attributed to the different brain biodistributions of these two formulations: most of the PLGA-PEG/F127 nanoparticles were retained in the brain capillaries where they would be cleared with continued blood circulation. Many of the PLGA-PEG/P80 nanoparticles were not vascular-associated and were present in the brain parenchyma. Prior cellular uptake studies using P80-modified PLGA nanoparticles indicate that the P80-modified formulation results in greater brain endothelial cell association [31]. This explanation is further supported by the mechanism of ApoE-mediated particle transport. ApoE adsorption to a nanoparticle surface can result in receptor-ligand interaction with LDL receptor and LRP, which mediates transport across brain endothelial cells where LRP receptors are highly expressed in brain tissue [28,31]. However, no to minimal intracellular uptake in the neurons or microglia of PLGA-PEG/P80 nanoparticles was observed. The mechanism of particle translocation from BBB interaction to neuron or microglia uptake remains unknown. In addition, the degree of ApoE enrichment on the nanoparticle surface has an important effect on distribution [49]. PLGA-PEG/P80 nanoparticles were emulsified in P80 solution rather than conjugated with P80, so the ApoE affinity of these nanoparticles may not be strong enough to impact cellular association with neurons after the particles have crossed the BBB. The animals used in this study were healthy animals with an intact BBB. Levels of ApoE in the term neonate compared to the adult are not well studied, which limits the ability to assess whether ApoE is present in a high enough quantity to interact with PLGA-PEG/P80 particles in animals of this age. Even if the P80-modified nanoparticles are injected into the carotid artery, which has significantly higher brain uptake compared with jugular vein injection, nanoparticle brain accumulation is still limited [38].

Due to the lack of availability of an accessible tail vein at this age in rodents and the higher bioavailability compared to the subcutaneous and oral routes, i.p. injection is a commonly used administration route for neonatal laboratory rodents. The main pathway for nanoparticles to enter the systemic circulation from the peritoneal cavity is via lymphatic uptake rather than diffusion [46]. Therefore, one critical parameter for nanoparticle absorption following i.p. injection is particle size as opposed to the amount of injected dose. We did not observe any significant differences among all tissues when administering different i.p. doses of PLGA-PEG particles in neonatal rats (Figure S2). Additionally, Panagi et al. showed that the blood clearance of PLGA-PEG nanoparticles was independent of dose [50]. However, the physiological status of an individual rat may affect nanoparticle absorption [51], which could alter uptake into the systemic circulation. Lastly, there are prior reports that the time to reach the maximum serum concentration may be influenced by the position of the i.p. injection, where the maximum concentration might vary two-fold between injection position above and below the transverse mesocolon [52]. Therefore, i.p. injection does introduce several challenges and potential limitations for reproducible and high-yield systemic nanoparticle delivery, even in the neonate.

To confirm that the quantitative measurements of the dye were not due to free dye in our tissue samples, we assessed the stability of the dye conjugation to PLGA-PEG. PLGA-PEG nanoparticles do not have electron-dense atoms, limiting the resolution of visualization in tissue samples through standard electron microscopy techniques [33,53]. Therefore, we used an indirect method to verify the dye-labeling stability of PLGA-PEG nanoparticles after crossing the peritoneal cavity. Compared with the unconjugated CF647 sample, serum extracts from CF647-labeled PLGA-PEG/F127 and PLGA-PEG/P80 injected rats showed no peak for free CF647 with a UV-Vis detector at 650 nm and a fluorescence detector for Ex/Em at 635 nm/665 nm. This indicates that there was no detectable free CF647 in the blood after i.p. injection, confirming that the bond between CF647 and polymer remained intact after partitioning into the lymphatic system and entering the systemic circulation.

As visualized by the TEM images and supported by differences in biodistribution in the brain, the surfaces of the PLGA-PEG/F127 and PLGA-PEG/P80 particles are likely different, while their bulk physicochemical properties are comparable. The scattered surfactant placement on the nanoparticles indicated by electron-dense regions in the TEM images suggests surfactant association after nanoparticle collection and washing. Electron-dense regions were nonuniform on the nanoparticle surface, which could impact absorption of blood proteins such as ApoE and subsequent interactions with the vascular endothelium. The kinetics of corona formation and the composition of the corona can be different when the surface composition of a nanoparticle changes [54], which can alter nanoparticle biodistribution and half-life [55,56]. Further studies will need to provide a quantitative analysis of the protein corona formation on PLGA-PEG nanoparticles as a function of surface composition, orientation, and distribution.

## 5. Conclusions

PLGA-PEG nanoparticles are promising drug delivery vehicles for the neonatal population. PK data can guide dosage determination for improving the biodistribution of nanoparticles in neonates. In this study, we showed that PLGA-PEG nanoparticles formulated with different surfactants had different PK parameters and biodistributions at the whole body and organ level. Compared with PLGA-PEG/P80, PLGA-PEG/F127 had a longer systemic circulation time and higher bioavailability, and thus had a longer half-life in the heart, lung, spleen, kidney, and brain. In the brain, PLGA-PEG/P80 had better distribution in the brain parenchyma, while PLGA-PEG/F127 remained associated with brain capillaries, although both formulations had low brain accumulation. The neonatal population is underrepresented in PK clinical trials, resulting in a data gap that is a prominent cause of off-label drug use in neonates. Understanding the PK profiles and biodistribution, as well as associated residence time, of PLGA-PEG and other polymer nanoparticles in neonates will help with the design and implementation of therapeutic nanoparticles with maximum efficiency and minimum toxicity. Neonatal PK data will help improve the speed of clinical translation and drug safety for the use of nanomedicines in this underserved population.

## Figures and Tables

**Table 1:** Physicochemical properties of PLGA-PEG/F127 and PLGA-PEG/P80. All values are reported as mean ± standard error of the mean (SEM) (n = 3).

Formulation	Size ± SEM (nm)	PDI ± SEM	Zeta Potential ± SEM (mV)
PLGA-PEG/F127	60.2 ± 0.8	0.2 ± 0.01	−2.6 ± 0.3
PLGA-PEG/P80	66.2 ± 1.4	0.2 ± 0.01	−2.0 ± 0.3

**Table 2:** PK parameters of PLGA-PEG/F127 and PLGA-PEG/P80 nanoparticles in P10 rat serum after i.p. injection (n = 4 per timepoint). *T_max_*, time to reach maximum concentration; *C_max_*, maximum concentration; *V_d_*, volume of distribution; *V_ss_*, volume of distribution at steady state; T_1/2_, half-life; AUC, area under the curve; MRT, mean residence time. *T_max_* is the experimental value, and all other PK parameters are reported as mean ± standard deviation.

Formulation	PK Parameter	
PLGA-PEG/F127	*T_max_* (h)	4
	*C_max_* (mg/mL)	2.1 ± 0.4
	*V_d_* (mL)	1.4 ± 0.4
	*V_ss_* (mL)	1.2 ± 1.1
	Clearance (mL/h)	0.17 ± 0.03
	T_1/2_ (h)	5.9 ± 2.1
	AUC (h-mg/mL)	23.1 ± 3.8
	MRT (h)	7.2 ± 1.7
PLGA-PEG/P80	*T_max_* (h)	4
	*C_max_* (mg/mL)	2.3 ± 0.6
	*V_d_* (mL)	0.74 ± 0.3
	*V_ss_* (mL)	1.6 ± 1.6
	Clearance (mL/h)	0.31 ± 0.06
	T_1/2_ (h)	1.7 ± 0.7
	AUC (h-mg/mL)	12.4 ± 2.3
	MRT (h)	5.2 ± 1.5

**Table 3:** PK parameters of PLGA-PEG/F127 and PLGA-PEG/P80 nanoparticles in P10 rat organs (heart, lung, liver, spleen, and kidney) after i.p. injection (n = 4 per timepoint). *T_max_*, time to reach maximum concentration; *C_max_*, maximum concentration; *V_d_*, volume of distribution; *V_ss_*, volume of distribution at steady state; T_1/2_, half-life; AUC, area under the curve; MRT, mean residence time. *T_max_* is the experimental value, and all other PK parameters are reported as mean ± standard deviation.

	Organ					
Formulation	PK Parameter	Heart	Lung	Liver	Spleen	Kidney
PLGA-PEG/F127	*T_max_* (h)	4	8	24	24	8
	*C_max_* (mg/mL)	0.19 ± 0.06	0.069 ± 0.04	1.2 ± 0.1	0.70 ± 0.2	0.19 ± 0.07
	*V_d_* (mL)	13.1 ± 6.9	591.8 ± 531.4	0.77 ± 0.1	5.3 ± 1.2	22.7 ± 6.4
	*V_ss_* (mL)	13.0 ± 15.5	33.5 ± 53.0	1.8 ± 1.2	2.9 ± 2.3	10.9 ± 9.7
	Clearance (mL/h)	1.0 ± 0.2	4.6 ± 2.3	0.082 ± 0.07	0.11 ± 0.2	0.47 ± 0.08
	T_1/2_ (h)	9.4 ± 2.4	89.3 ± 21.0	6.5 ± 0.5	33.5 ± 2.9	33.4 ± 6.7
	AUC (h-mg/mL)	4.0 ± 1.0	0.84 ± 0.4	47.0 ± 4.1	34.9 ± 4.8	8.2 ± 1.3
	MRT (h)	13.4 ± 6.3	7.3 ± 5.4	22.0 ± 2.7	26.0 ± 4.9	23.2 ± 5.3
PLGA-PEG/P80	*T_max_* (h)	4	8	24	8	24
	*C_max_* (mg/mL)	0.12 ± 0.04	0.025 ± 0.01	0.89 ± 0.1	0.53 ± 0.1	0.065 ± 0.02
	*V_d_* (mL)	15.7 ± 12.5	114.8 ± 107.0	2.1 ± 0.6	7.4 ± 2.9	48.1 ± 22.0
	*V_ss_* (mL)	28.2 ± 41.2	96.8 ± 152.0	2.5 ± 2.3	4.1 ± 4.3	27.3 ± 30.8
	Clearance (mL/h)	2.2 ± 0.8	9.9 ± 3.8	0.11 ± 0.01	0.24 ± 0.05	1.4 ± 0.4
	T_1/2_ (h)	4.9 ± 2.3	8.0 ± 1.8	13.5 ± 2.4	21.2 ± 4.1	24.0 ± 2.2
	AUC (h-mg/mL)	1.7 ± 0.6	0.39 ± 0.2	36.3 ± 4.9	15.8 ± 3.2	2.8 ± 0.7
	MRT (h)	12.6 ± 9.0	9.7 ± 8.3	24.1 ± 6.3	17.0 ± 5.7	19.6 ± 7.3

**Table 4:** PK parameters of PLGA-PEG/F127 and PLGA-PEG/P80 nanoparticles in P10 rat brain after i.p. injection (n = 4 per timepoint). *T_max_*, time to reach maximum concentration; *C_max_*, maximum concentration; *V_d_*, volume of distribution; *V_ss_*, volume of distribution at steady state; T_1/2_, half-life; AUC, area under the curve; MRT, mean residence time. *T_max_* is the experimental value, and all other PK parameters are reported as mean ± standard deviation.

Formulation	PK Parameter	
PLGA-PEG/F127	*T_max_* (h)	4
	*C_max_* (mg/mL)	0.013 ± 0.01
	*V_d_* (mL)	261.5 ± 281.7
	*V_ss_* (mL)	208.0 ± 256.4
	Clearance (mL/h)	13.6 ± 7.2
	T_1/2_ (h)	13.3 ± 4.4
	AUC (h-mg/mL)	0.28 ± 0.2
	MRT (h)	15.3 ± 14.4
PLGA-PEG/P80	*T_max_* (h)	4
	*C_max_* (mg/mL)	0.020 ± 0.01
	*V_d_* (mL)	38.7 ± 38.5
	*V_ss_* (mL)	379.2 ± 628.2
	Clearance (mL/h)	16.7 ± 8.8
	T_1/2_ (h)	1.6 ± 0.9
	AUC (h-mg/mL)	0.23 ± 0.1
	MRT (h)	22.7 ± 19.1

## Data Availability

The data can be provided upon request to the author.

## Supplementary Materials

The following supporting information can be downloaded at: https://www.mdpi.com/article/10.3390/pharmaceutics15041176/s1, Figure S1: Representative HPLC chromatograms for CF647 dye and particle serum samples; Figure S2: Dose-dependent biodistribution of PLGA-PEG/P80.
