# Supplementary material for: Neonatal Pharmacokinetics and Biodistribution of Polymeric Nanoparticles and Effect of Surfactant
Source: Pharmaceutics. 2023 Apr 7;15(4):1176. doi: 10.3390/pharmaceutics15041176 (PMC10140984; doi:10.3390/pharmaceutics15041176)
Supplement: Supplementary file 1 [file pharmaceutics-15-01176-s001.zip › pharmaceutics-2254967-supplementary.pdf]

Neonatal pharmacokinetics and biodistribution of polymeric nanoparticles and effect of surfactant

Nuo Xu[1], Megan Wong[1], Gabrielle Balistreri[2], Elizabeth Nance[1-4,]*

[1]Department of Chemical Engineering, University of Washington, Seattle WA 98195

[2]Molecular Engineering & Sciences Institute, University of Washington, Seattle, WA 98195, USA

[3]Department of Bioengineering, University of Washington, Seattle, Washington 98195

[4]Center for Human Development and Disability, University of Washington, Seattle, Washington, 98195

*Corresponding author: eanance@uw.edu

Supplemental figure

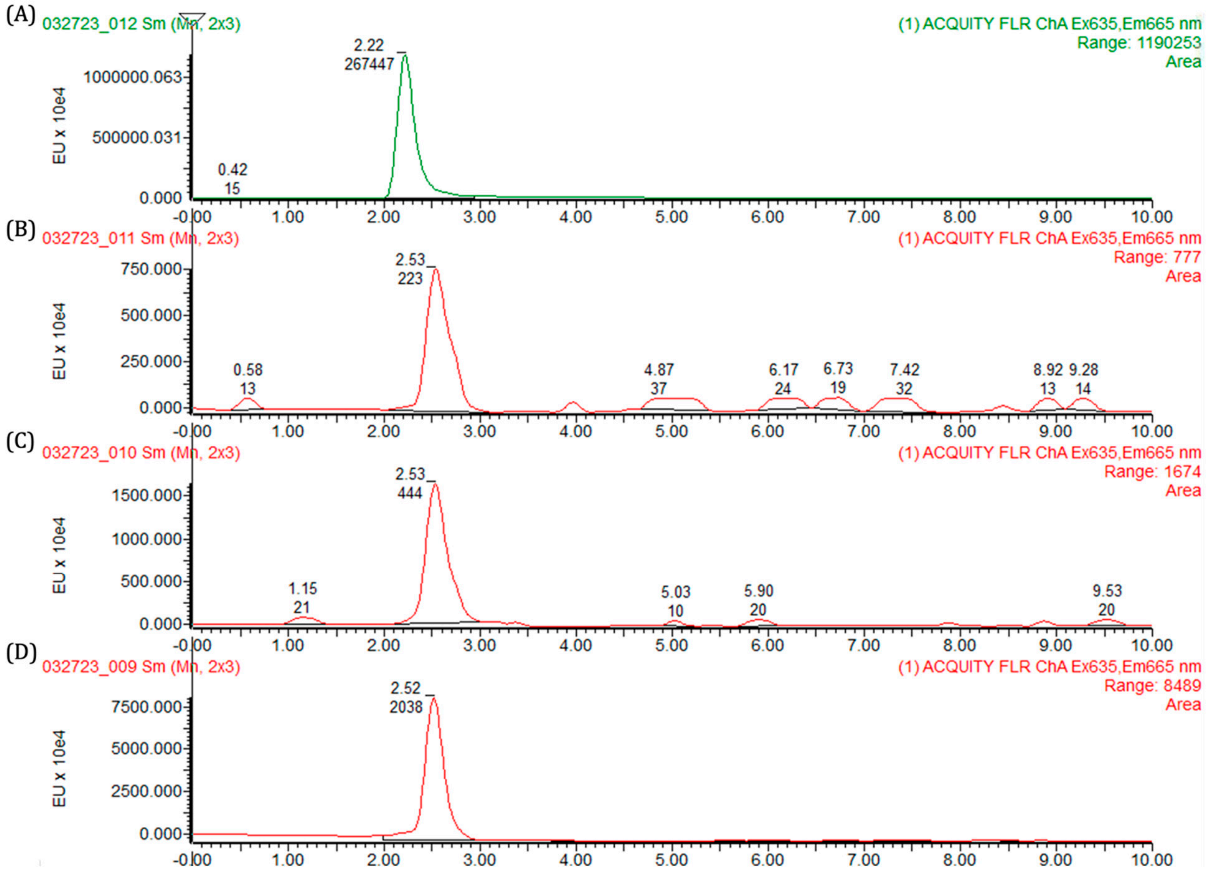

Figure S1. Representative HPLC chromatograms acquired based on Ex/Em at 635 nm/665 nm for: (A) Free CF647 dye at a 20 nmol/mL concentration, (B) blank serum extracts, (C) serum extracts obtained from rat 4 h post injection of PLGA-PEG/F127 at a 150 mg/kg dosage, and (D) serum extracts obtained from rat 4 h post injection of PLGA-PEG/P80 at a 150 mg/kg dosage. The retention time for the serum extracts is different than the free CF647, indicating that there was no CF647 detected in serum extracts.

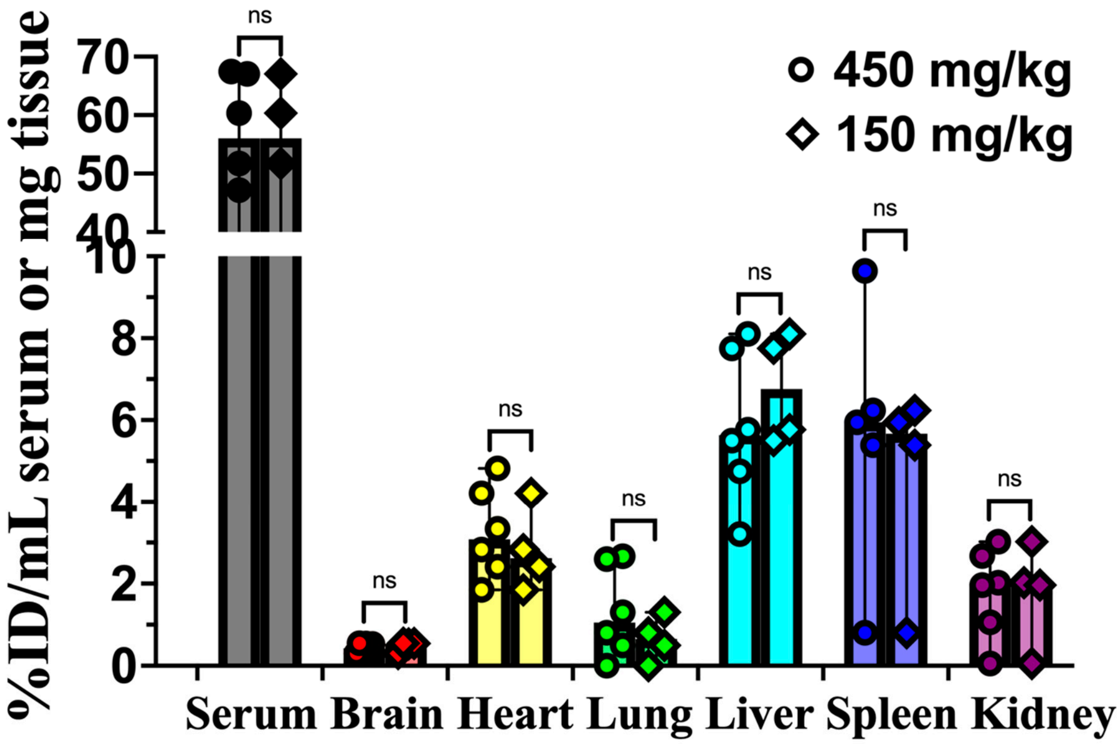

Figure S2. Dose-dependent biodistribution of PLGA-PEG/P80. Biodistribution of PLGA-PEG/P80 at 4 h after i.p. injection with 450 mg/kg i.p. dosage versus 150 mg/kg i.p. dosage in healthy P10 rat (n≥4 per timepoint). Graph displays median ± 95% confidence interval. Group differences were evaluated by Welch's t-test, ns: not significant.
